# Hospitals and Income Tax

**Published:** 1924-05

**Authors:** Joseph E. Stone

**Affiliations:** Accountant of St. Thomas's Hospital


### HOSPITALS AND INCOME TAX.

By JOSEPH E. STONE, F.S.A.A. (Hons.), F.S.S., F.R.Econ.S., Accountant of St. Thomas's Hospital.

HOSPITALS are exempt from the payment of tax upon income. Section 30 (1) of the Finance Act, 1921, provides that exemption shall be granted from Income Tax under Schedule A in respect of lands, tenements, &c., owned and/(or) occupied by a charity; from income tax under Schedule B in respect of lands occupied by a charity; (the exemption does not apply in the case of lands which are occupied for the purpose of husbandry unless the work is mainly carried on by beneficiaries of the charity and the profits, if any, arising therefrom are applied solely to the purposes of the charity); and from income tax under Schedule D in respect of the profits of a trade carried on by any charity if the work in connection with the trade is mainly carried on by beneficiaries, provided that the rents and interest which would otherwise have been taxable are properly applied to charitable purposes. Thus, where a hospital is in receipt of income from any of the foregoing sources and is received less income tax, the tax so deducted may be recovered. It should at once be noted, however, that the exemption does not extend to tax in respect of any rent payable or other annual payment to be made by a charity in respect of lands, tenements, etc., which are in the use and enjoyment of a person with a total annual income amounting to £150 or more. The expression "charity" for the purposes of income tax means any body of persons or trust established for charitable purposes only. It has, therefore, no necessary connection with the poor.

### Limitation of Exemption.

To obtain the benefit of exemption, a hospital must be maintained wholly or in part by charity. In Needham *v.* Bowes it was held that an institution founded by charitable donations, but supported wholly out of payments by the patients, of whom some paid more and some paid less than the cost of their maintenance, was not exempt from tax under Schedule A as a "hospital," the exemption being restricted to a hospital maintained wholly or in part by charity. The case of St. Andrew's Hospital *v.* Shearsmith was decided similarly on the same grounds.

### Hospitals Which are Self-Supporting.

In Cawse (Surveyor of Taxes) *v.* Committee of Northampton Lunatic Hospital, it was sought to exclude the hospital (which was originally built out of charitable funds and a portion of the income of which was derived from interest on the Endowment Fund) from the benefit of exemption from tax under Schedule A and Inhabited House Duty on the ground that, owing to the receipts for the care of patients who paid on a higher than the ordinary rate, the hospital was self-supporting. The Committee contended that the word "profits" in the Income Tax Acts means what remains after deducting the value of the occupation of the premises where the profit is made, and that if this were done there would be a loss instead of a profit. Further, that the mere fact that in a single year the hospital had been self-supporting did not disentitle them to exemption; the whole of the income was by the trust deeds to be applied to charitable purposes only. It was held that the Committee were entitled to the exemption claimed.

### Profits from Trading.

The profits derived from the carrying on of any kind of trade by a hospital are not exempt from income tax, no matter how they are applied. Such trades would include homes for paying patients, letting of rooms, etc. This follows from the decision in Cowan *v.* Governors of the Rotunda Hospital. In this case the hospital owned rooms which were let out for meetings, the surplus income after all expenditure had been met being applied to the maintenance of the hospital. A considerable sum was derived from these lettings, and it was held that the hospital was carrying on a trade, that the profits were outside the scope of Schedule A, and that they were assessable under Schedule D. It should be particularly noted that this decision only applies to cases where the hospital itself is carrying on the trade. It does not apply to a hospital which merely receives from trustees an annual payment from the profits of a business bequeathed to the hospital but carried on by the trustees on their behalf (Trustees of the Shaftesbury Homes and the Arethusa Training Ship, on appeal).

### Allocation of Income.

An important case in connection with charitable bequests and repayment of income tax was that of Dr. Barnardo's Homes *v.* Special Commissioners, which was decided by the House of Lords. In this case the charity had received a share of the residuary estate under a will. The testator died in 1914 but, owing to disputes, the residue was not actually ascertained until 1916, and it therefore included accumulations of income which had borne tax by deduction. The charity claimed that tax on this accumulated income should be repaid to them, but the House of Lords held that a legatee of a share of the residue had no interest in the estate until the residue is ascertained, on the ground that residuary estate is in a different position from specific legacies which date back to the death of the testator. The repayment was thus refused, and the share of the residue, plus the accumulated income, treated as one capital sum. This decision, however, does not hold good to-day, and Section 30 (1) of the Finance Act, 1922, expressly provides that the accumulations of income shall be exempt from income tax.

### Officers' Quarters.

Such parts of a hospital as are occupied by officers of the hospital in receipt of an income of £150 or more per annum are not exempt from assessment under Schedule A (Schedule A, No. VI., Rule I). This question of liability to tax under Schedule A in respect of such quarters was raised in Governors of Sutton's Hospital in Charterhouse *v.* Elliott.

The charity scheme provided that certain officials, such as Registrar, Receiver, and Chaplain, should have the use and occupation of their living quarters at the hospital free of rent, rates and taxes. (No. VI. of Schedule A exempts property belonging to hospitals, etc., but specifically leaves in charge the annual value of quarters as above.) The assessment was made on the charity as occupiers. This follows from Tennant *v.* Smith, where it was held that an occupation by an employee, where enjoined on him by the employer, was an occupation by the employer. The charity claimed that as the officials were not technically the "occupiers," and as Schedule A is only assessable upon the occupier, the officials' quarters should not be charged on the charity, as a charity is treated as being exempt from such tax, and that the tax could not be charged on the officials. They claimed that the assessment should be discharged. The Court, however, decided in favour of the Crown, holding that the charity were the occupiers, and that under No. VI., Schedule A, the assessment was rightly made.

Where officials holding an office, etc., are required by statute to make contributions towards superannuation allowances or gratuities on retirement or death, these contributions are allowable deductions for assessment from their salaries, etc., for the year. But where any such contributions are subsequently repaid, tax is deductable from the repayment equal to tax (on principal and interest, if any) that would have been paid throughout if there had been no tax allowance (Finance Act, 1922, Section 31).

